# Factors Influencing Telehealth Implementation and Use in Frontier Critical Access Hospitals: Qualitative Study

**DOI:** 10.2196/24118

**Published:** 2021-05-05

**Authors:** Saira Naim Haque, Sydney DeStefano, Alison Banger, Regina Rutledge, Melissa Romaire

**Affiliations:** 1 RTI International Research Triangle Park, NC United States

**Keywords:** telehealth, rural health, health IT, telemedicine, virtual care

## Abstract

**Background:**

Telehealth has potential to help individuals in rural areas overcome geographical barriers and to improve access to care. The factors that influence the implementation and use of telehealth in critical access hospitals are in need of exploration.

**Objective:**

The aim of this study is to understand the factors that influenced telehealth uptake and use in a set of frontier critical access hospitals in the United States.

**Methods:**

This work was conducted as part of a larger evaluation of a Centers for Medicare & Medicaid Services–funded demonstration program to expand cost-based reimbursement for services for Medicare beneficiaries for frontier critical access hospitals. Our sample was 8 critical access hospitals in Montana, Nevada, and North Dakota that implemented the telehealth aspect of that demonstration. We reviewed applications and progress reports for the demonstration program and conducted in-person site visits. We used a semistructured discussion guide to facilitate conversations with clinical, administrative, and information technology staff. Using NVivo software (QSR International), we coded the notes from the interviews and then analyzed the themes.

**Results:**

Several factors influenced the implementation and use of telehealth in critical access hospitals, including making changes to workflow and infrastructure as well as practitioner acceptance and availability. Participants also cited technical assistance and support for implementation as supportive factors.

**Conclusions:**

Frontier critical access hospitals may adopt telehealth to overcome challenges such as distance from specialty practitioners and workforce challenges. Telehealth can be used for provider-to-patient and provider-to-provider interactions to improve access to care, remove barriers, and improve quality. However, the ability of telehealth to improve outcomes is limited by factors such as workflow and infrastructure changes, practitioner acceptance and availability, and financing.

## Introduction

Telehealth has potential to help individuals in rural areas overcome geographical barriers and to improve access to care. Barriers to access include travel time, transportation, cost, and other logistical considerations, which telehealth can help address [1]. Access to care can be enhanced through telehealth to connect practitioners and patients who are not co-located, which enables patients to receive care that is not otherwise available locally.

Improvements in outcomes through telehealth largely occur through improved management of chronic disease and improved follow-up [2,3]. Many rural areas are not adequately served by practitioners; therefore, routine follow-up can involve extensive travel, resulting in delays in receiving care, missed appointments, and other issues [4,5]. Telehealth can promote improved adherence to care plans and can contribute to improved outcomes and reduced costs through averted hospital stays and emergency department visits [3,6].

Although telehealth use has become more prevalent, prior to the COVID-19 pandemic, it was not widespread; thus, its benefits have not been fully realized [7-9]. For telehealth to show potential benefits in the long term beyond the public health emergency, its services must fit into changes in the health care landscape, including the advent of alternative care delivery and payment models, greater integration of physical and behavioral health services, and efforts to address workforce challenges [10,11].

To improve uptake of telehealth in communities where telehealth may be most needed and where benefits to patient access to health care may be significant, policy makers have turned to critical access hospitals (CAHs) because CAHs are charged with providing health care in rural communities that would otherwise not have basic inpatient, outpatient, and emergency care services. Recognizing the central role that CAHs play in integrating and providing access to health care in rural communities, the Centers for Medicare & Medicaid Services (CMS) implemented the 3-year Frontier Community Health Integration Project (FCHIP) Demonstration. FCHIP addressed four service areas—telehealth, skilled nursing facility care, home health services, and ambulance services—in 10 CAHs in Montana, Nevada, and North Dakota [2]. These frontier areas are characterized as having fewer than 10 people per square mile and are predominantly located in the western part of the United States.

This article reports on the qualitative findings for the first year of the telehealth component of the demonstration. We sought to identify and describe barriers and facilitators influencing telehealth adoption for CAHs. These findings can inform other telehealth efforts in rural areas.

## Methods

This study was conducted as part of an evaluation of the FCHIP Demonstration project, which includes telehealth as a component. The overall study received approval from the Research Triangle Institute Institutional Review Board. Primary and secondary data on hospital landscape and FCHIP implementation activities were collected through in-person site visits and demonstration-related document review for each of the participating FCHIP CAHs. Site visit data collection included key informant interviews with a variety of stakeholders at each CAH.

### Site Visits

Researchers with expertise in rural health and health informatics conducted on-site interviews with each CAH during June and July of 2017. We identified key stakeholders at each of the sites and interviewed this convenience sample, which included hospital leadership (eg, hospital administrators, medical directors), practitioners, and administrative support staff. We developed discussion guides for each category of informant. Discussion topics included any changes made (eg, staffing, infrastructure), implementation progress, perceived impacts, and any facilitators and barriers to implementation. We conducted interviews with 36 individuals at 8 CAHs participating in the telehealth component of FCHIP. Two FCHIP-participating CAHs focused demonstration activities on service areas other than telehealth. We were not able to secure interviews with patients or caregivers.

### Document Review

The evaluation team collected information about each CAH from documents that the CAH completed as part of the evaluation, including applications, progress plans, and facility overview/quarterly reports. These reports were submitted to CMS, which shared them with the evaluation team. These documents were used to triangulate with the interview findings.

### Data Analysis

This work is part of a larger evaluation to understand the impact of the FCHIP Demonstration on participating CAHs. The evaluation focused on how the FCHIP Demonstration can improve access to care, integration, and delivery. We synthesized the qualitative information from site visits and the document review. We developed a codebook based on the literature and the overall goals of the evaluation. Items in the codebook included workflow, practitioner acceptance, infrastructure, and financing. We then coded data using NVivo (QSR International), a qualitative software analysis package. Next, we developed key themes using discussion topics from the interview protocols, the document reviews, and overall evaluation goals. Finally, we generated reports by each theme for review and analysis.

## Results

CAHs expanded their capabilities to provide different types of services via telehealth with the hope that these new services would allow more community members to remain in the local community for care. Factors that influenced telehealth implementation and use include workflow, practitioner availability and acceptance, infrastructure and cost, and sustainability.

### Workflow

Workflow changes included adding telehealth to staff responsibilities, education, training, and outreach. To implement or expand telehealth services, CAHs added new responsibilities to existing staff members’ workloads; none of the CAHs reported hiring new staff for telehealth. New responsibilities included setting up equipment, managing referrals, and coordinating between CAHs and distant providers. Staff who assumed these new duties were nurses, administrative support staff, ward clerks, and medical assistants.

The addition of responsibilities was predicated on training. For example, staff received training on how to use equipment, document telehealth encounters for reimbursement, and incorporate telehealth encounters into the workflow. Providing outreach to surrounding communities was an additional responsibility for CAH staff. Outreach included explaining how telehealth worked, demonstrating the value of telehealth, and overcoming resistance to change in referring and using telehealth services. Outreach to communities included different audiences, such as practitioners and the general community.

Practitioners in the frontier community who provide referrals to telehealth services, distant providers who deliver services, and staff who support them also received training and outreach to improve telehealth acceptance so they could change their workflows to accommodate telehealth. Interviewees had mixed feelings on training, indicating that training on using telehealth technologies and working with partners providing distant services ranged from straightforward to onerous. As one CAH administrator indicated:

Being able to help patients is fabulous. As far as our staff go, it is a learning curve. We are all new to what we need to be doing. … Now we have streamlined … Now we have a system that works.

Staff at one site described conducting outreach to encourage telehealth referrals. Rather than waiting for practitioners to refer patients for telehealth services, the site directed staff to take a more active role in telehealth decisions. The staff started asking patients if they would be interested in telehealth services, then called referring practitioners to see whether telehealth would be appropriate. Staff required some training on making calls, referring practitioners, and engaging in patient outreach.

### Practitioner Availability and Acceptance

Sites participating in telehealth noted that administrative barriers presented the greatest challenges during the demonstration. Several sites noted that they were not able to provide services due to impediments to securing properly credentialed practitioners with availability. These sites faced challenges in obtaining the proper state credentials that give distant providers the necessary privileges to provide services for CAH patients. Furthermore, practitioners and administrators described efforts to use telehealth to provide specialty care, particularly dermatology and behavioral health services, but were told that distant providers were at capacity and were not accepting any more patients through telehealth.

Additionally, multiple sites noted that telehealth service provision was outside the usual scope of work for many CAH practitioners and staff and was often met with reluctance to change. Using telehealth requires a referral and additional paperwork; thus, some medical directors preferred their historical approach of calling a specialist colleague (by telephone) to obtain immediate answers. Participants indicated that having a practitioner champion was key to increasing volume because practitioners make telehealth referrals. As one participant indicated, “Providers are the big issue of adopting telehealth because they have to give the referrals.” This challenge highlights the importance of practitioner outreach as a facilitator so that telehealth services are available to patients.

### Infrastructure

Telehealth implementation required a few infrastructure and operational changes, which could be barriers to implementation and use. Infrastructure modification included technical and physical changes. Some sites modified their electronic health records (EHRs) to provide a consistent mechanism to document telehealth services. Other sites required equipment and physical changes. For example, some sites purchased mobile telehealth equipment so that they did not have to share equipment between the CAH-owned clinic and the CAH itself.

### Cost and Sustainability

Hospital staff reported that upfront costs for equipment combined with very low use would likely lead to a negative impact on financial performance. CAH staff members noted the high cost to procure the necessary telehealth equipment using their regular operating budget and expressed a desire for alternative sources of funding for this upfront cost. At hospitals that invested in infrastructure and equipment, it was noted that an increase in volume was needed to obtain sufficient reimbursements to offset the investments and improve financial performance.

Hospital staff reported varied experiences in billing for telehealth services. One hospital administrator mentioned that she was given a list of Current Procedural Terminology codes for Medicare telehealth claims and was able to use them to bill for these services easily. However, a staff member at another site noted that their EHR system was not equipped to properly document the service dates and subsequently bill for telehealth services. Although one hospital administrator felt that Medicare billing guidance was straightforward, some felt that guidance on Medicaid billing was lacking or complex, which led to confusion when billing for services.

## Discussion

### Principal Findings

CAH staff identified several obstacles to telehealth: difficulty establishing the needed relationship with appropriate distant providers due to credentialing issues, capacity limits of distant providers, potential distant providers in different health care networks, and distant providers unwilling to use telehealth. Another barrier was unwillingness to integrate telehealth into care delivery by referring providers in the patients’ communities. Contextual factors such as accommodating process flows of local practitioners, patients, and staff was another barrier. Limited resources to support telehealth was another environmental factor that influenced telehealth uptake. Although the clinical staff we interviewed recognized the benefits of telehealth, they also indicated that workflow changes were needed so that practitioners would refer to telehealth services rather than face-to-face encounters and incorporate telehealth into their clinical and administrative practices.

### Factors Influencing Telehealth Uptake and Use

#### Staff Acceptance

Telehealth represents a change in delivery mechanism and thus requires acceptance by practitioners, staff, and patients alike. CAH staff indicated that because practitioners are the gatekeepers who provide referrals to telehealth services, their engagement was critical to telehealth uptake, which was consistent with other findings [12]. In addition, if staff were engaged with telehealth, they were more likely to serve as champions internally and share their excitement with patients, which also resonates with findings from the literature [13-16]. Administrators across CAHs indicated the importance of getting practitioners and staff excited about and engaged with telehealth. Engagement was a key success factor.

#### Operational Considerations

Telehealth implementation reflects an organizational shift that requires some operational changes including clinical and administrative workflows, relationships with distant providers, and technical considerations [17]. Frontier CAHs generally seek specialty services and support from distant providers. Thus, operationalizing the relationship with distant providers requires structure that must be in place before implementation [4,18]. Staff at frontier CAHs indicated that ensuring that their providers could easily refer patients to distant specialty providers was a key success factor for telehealth uptake. Operational considerations also include items for hospital leadership, such as credentialing distant providers so that they can provide services at the facility [19]. The CAH staff indicated that credentialing of providers was a significant barrier.

In addition to relationships between providers, workflow considerations include ensuring that services appear seamless to the patient [20]. CAHs must identify processes (that may change ahead of time) so that policies are not developed on an ad hoc basis [21]. CAH staff indicated that they need implementation support to help identify what items needed to be changed and materials such as implementation guides to support telehealth implementation. Processes to consider include identifying and scheduling patients [18], making referrals and sharing information, and consenting. CAH staff reported that spending time on these processes helped facilitate care coordination.

#### Financial Considerations

Implementing and using telehealth involves financial considerations, primarily expenses due to infrastructure, staffing, and training, and offsetting those expenses with reimbursement. In addition, costs must be allocated for education, outreach, and workflow integration efforts. Although reimbursement for telehealth services is increasing, it still varies widely and is inconsistent [12,13,22,23]. CAH staff reported needing assistance with revenue cycles and with billing for services under the demonstration, particularly when they had not previously done so. Other financial considerations included structuring arrangements with distant providers. Telehealth involves sharing patients with practitioners who are in different locations, and CAHs needed assistance with billing processes to ensure payment for services [4,5,24].

### Limitations

Some limitations may have affected this work. First, our major data source for this report was the responses that the authors collected during key informant interviews. The number of interviewees was small, which limits generalizability. Our secondary data source was reports, although many of these were developed by the same people we interviewed. Although the goal of the interviews was to obtain feedback (including viewpoints) from a variety of stakeholders, there is no guarantee that the individuals who participated in the interviews are representative of the entire staff at the CAHs. In addition, some of the interviewees were responsible for creating some of the reports reviewed in the document review. Therefore, the analysis results from the qualitative data may not represent all perspectives.

Furthermore, this work focuses on a small number of participating CAHs (n=8) in three states. Each of these CAHs was in a designated frontier area; therefore, the population was less dense than is generally found in rural areas. Each CAH has different internal and community resources available that may affect the successful implementation of telehealth. In addition, all of the CAHs were in the western part of the country. These factors may impact generalizability to other rural areas.

### Conclusion

Frontier CAHs face challenges, including distance and provider availability, that telehealth implementation and use could address. Telehealth can be used for provider-to-patient and provider-to-provider interactions. To realize the promise of telehealth, frontier CAHs need support to improve factors that influence implementation and use.

During the COVID-19 pandemic, many organizations moved to telehealth very rapidly [25]. Although many of the initial increases in telehealth uptake were not sustained, telehealth encounters are above prepandemic levels [26,27]. Thus, understanding barriers and facilitators to telehealth generally can be useful for postpandemic planning. Across CAHs, support needs include change management, service identification for the community, credentialing, and reimbursement. Factors such as necessary workflow and infrastructure changes, practitioner acceptance and availability, and financing must be addressed to improve telehealth uptake. Supporting telehealth includes organizational and policy-level changes that can increase access and improve outcomes in rural communities.

## Abbreviations

CAH: critical access hospital
CMS: Centers for Medicare & Medicaid Services
EHR: electronic health record
FCHIP: Frontier Community Health Integration Project
